# Defining and Evaluating a Core Genome Multilocus Sequence Typing Scheme for Whole-Genome Sequence-Based Typing of *Klebsiella pneumoniae*

**DOI:** 10.3389/fmicb.2017.00371

**Published:** 2017-03-08

**Authors:** Haijian Zhou, Wenbing Liu, Tian Qin, Chen Liu, Hongyu Ren

**Affiliations:** ^1^State Key Laboratory for Infectious Disease Prevention and Control, National Institute for Communicable Disease Control and Prevention, Chinese Centre for Disease Control and PreventionBeijing, China; ^2^Collaborative Innovation Centre for Diagnosis and Treatment of Infectious DiseasesHangzhou, China; ^3^Novogene Bioinformatics Technology Co. LtdBeijing, China

**Keywords:** core genome multilocus sequence typing, *Klebsiella pneumoniae*, whole-genome sequence, outbreak investigation, population structure analysis

## Abstract

At present, the most used methods for *Klebsiella pneumoniae* subtyping are multilocus sequence typing (MLST) and pulsed-field gel electrophoresis (PFGE). However, the discriminatory power of MLST could not meet the need for distinguishing outbreak and non-outbreak isolates and the PFGE is time-consuming and labor-intensive. A core genome multilocus sequence typing (cgMLST) scheme for whole-genome sequence-based typing of *K. pneumoniae* was developed for solving the disadvantages of these traditional molecular subtyping methods. Firstly, we used the complete genome of *K. pneumoniae* strain HKUOPLC as the reference genome and 907 genomes of *K. pneumoniae* download from NCBI database as original genome dataset to determine cgMLST target genes. A total of 1,143 genes were retained as cgMLST target genes. Secondly, we used 26 *K. pneumoniae* strains from a nosocomial infection outbreak to evaluate the cgMLST scheme. cgMLST enabled clustering of outbreak strains with <10 alleles difference and unambiguous separation from unrelated outgroup strains. Moreover, cgMLST revealed that there may be several sub-clones of epidemic ST11 clone. In conclusion, the novel cgMLST scheme not only showed higher discriminatory power compared with PFGE and MLST in outbreak investigations but also showed ability to reveal more population structure characteristics than MLST.

## Introduction

*Klebsiella pneumoniae* is associated with a wide range of diseases, including urinary tract infections, respiratory tract infections, septicemia, pyogenic liver abscess, primary osteomyelitis and so on (Podschun and Ullmann, 1998; Brisse et al., 2009; Prokesch et al., 2016). This organism is considered as an opportunistic pathogen which commonly causes nosocomial infections (Podschun and Ullmann, 1998; Pitout et al., 2015; Campos et al., 2016). Nosocomial isolates of *K. pneumoniae* often display multidrug-resistance phenotypes, making difficulty in choosing sensitive antibiotics for treatment (Keynan and Rubinstein, 2007; Lee et al., 2016). The emergence and rapid spread of drug-resistant *K. pneumoniae* isolates is becoming a serious antibiotic management problem and causing great concern worldwide.

A large number of subtyping techniques have been used to identify clusters and outbreaks, trace the source and determine the spreading chain in the investigation of *K. pneumonia* infections or outbreak and to study the population structure and pathogen evolution of *K. pneumonia* (Podschun and Ullmann, 1998). Among these techniques, multilocus sequence typing (MLST) and pulsed-field gel electrophoresis (PFGE) are the two most frequently used methods to investigate outbreaks of *K. pneumoniae* infection (Jin et al., 2015; Mshana et al., 2015; Zhu et al., 2016). PFGE has the ability to definite and distinguish outbreak and non-outbreak isolates and PFGE data can be exchanged between the different labs (Swaminathan et al., 2001). However, the operation of PFGE is cumbersome and this method typically takes several days to obtain results. The *K. pneumoniae* MLST scheme was developed in 2005 and then has been widely used to characterize diversity and epidemiology of *K. pneumoniae* isolates (Diancourt et al., 2005). MLST is a powerful method based on the sequencing of seven gene loci and a large MLST database is available. However, the discriminatory power of MLST could not meet the need for distinguishing outbreak and non-outbreak isolates (Jiang et al., 2015; Baraniak et al., 2016). So in outbreak investigations, both PFGE and MLST are needed to be carried out to meet all kinds of needs. It is a time-consuming and labor-intensive process. Recently, methods based on whole-genome sequencing were used to subtype and study the evolution of hypervirulent *K. pneumoniae* clones and showed a good typing ability (Bialek-Davenet et al., 2014; Struve et al., 2015).

A possible solution to this problem has been suggested by extending the MLST concept to the genome level based on whole-genome sequence data and comparison to a set of loci (e.g., the genes of the core genome) and allele variants indexed (Jolley and Maiden, 2010; Maiden et al., 2013). This method is known as core genome multilocus sequence typing (cgMLST). The cgMLST showed good typing ability of several pathogenic bacteria including *Neisseria meningitidis* (Bratcher et al., 2014), *Listeria monocytogenes* (Ruppitsch et al., 2015), *Mycobacterium tuberculosis* (Kohl et al., 2014), Methicillin-resistant *Staphylococcus aureus* (MRSA) (Leopold et al., 2014), *Legionella pneumophila* (Moran-Gilad et al., 2015), *Enterococcus faecium* (de Been et al., 2015), and *K. pneumoniae* (Bialek-Davenet et al., 2014). cgMLST is expected to be the main advantages of higher discriminatory power than MLST and easier to obtain and compare results than PFGE as it is based on sequencing. In this study we developed a cgMLST typing scheme for *K. pneumoniae* and then we evaluated this scheme for ability in outbreak investigations using isolates from one outbreak and several sporadic cases.

## Materials and methods

### Sequenced strains and analyzed whole genome sequences

**Figure 1:**
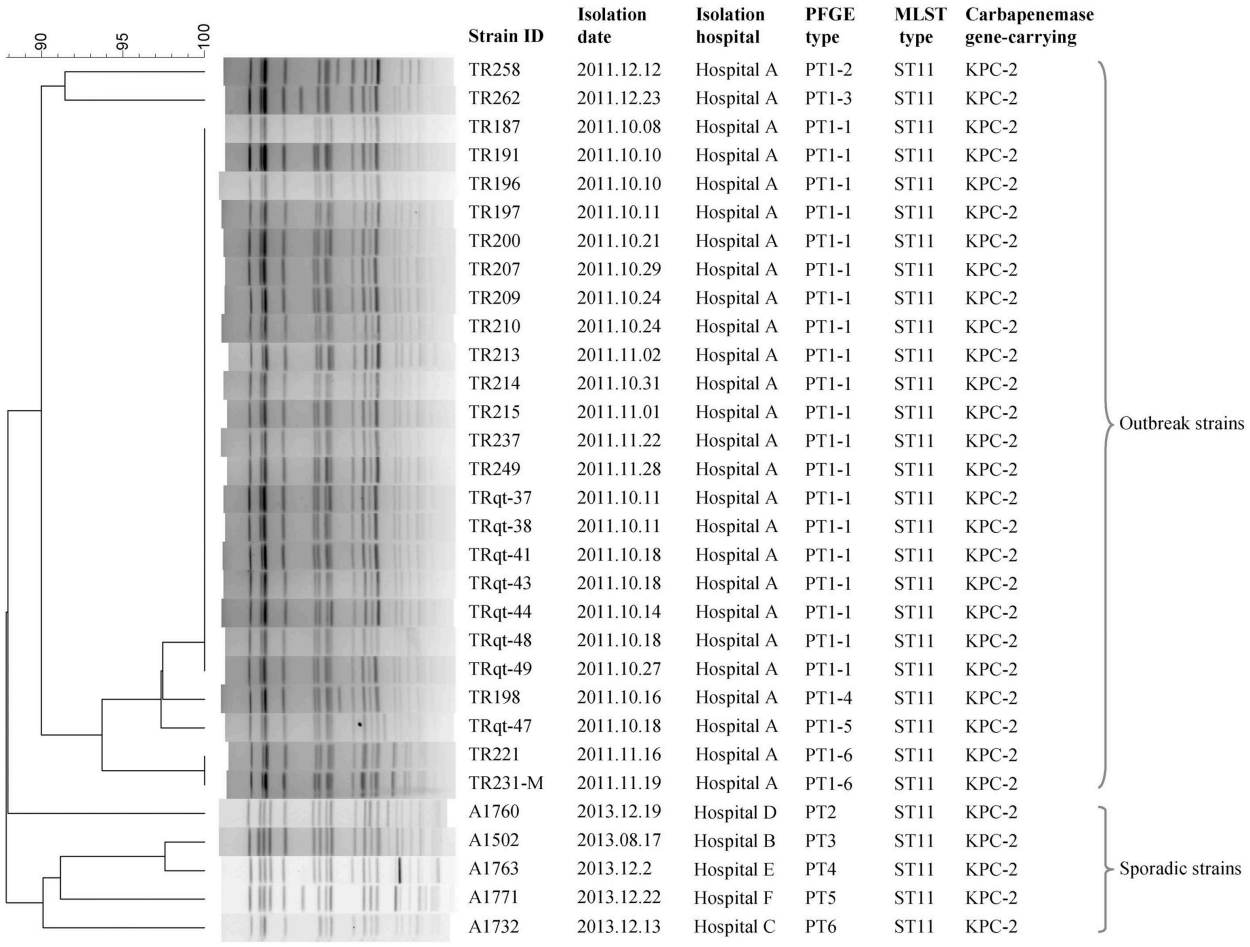
**PFGE and MLST subtyping of 31 ***K. pneumoniae*** strains sequenced in this study**.

The whole genome sequences of 31 *K. pneumoniae* strains were sequenced in this study (Table 1). These strains were isolated from one outbreak of *K. pneumoniae* infection and five sporadic cases. The outbreaks were identified by both epidemiological information data and molecular subtyping data (PFGE, MLST) (Figure 1). The sporadic cases were defined as no direct epidemiological relevance with any other case. A total of 907 whole genome sequences of *K. pneumoniae* obtained from the NCBI database (http://www.ncbi.nlm.nih.gov/) (download in January 7, 2016) were used in this study to screen the cgMLST target genes. The complete genome of *K. pneumoniae* strain HKUOPLC (GenBank assembly accession number GCA_001280925.1) was used as the reference genome to determine cgMLST target genes. All the *K. pneumoniae* whole genome sequences (except fastq data) released to NCBI database before January 7, 2016 were used in this study.

**Table 1:** **Characteristics of ***K. pneumoniae*** strains sequenced in this study**.

**Strain ID**	**Isolate origin**			**Molecular subtype**		**Genome sequencing results**		
	**Isolation date**	**Isolation hospital**	**Outbreak or sporadic**	**PFGE type**	**MLST type**	**No. of reads**	**Genome length (Mbp)**	**Coverage (%)**
TR196	2011.10.10	Hospital A	Outbreak	PT1-1	ST11	5064323	5,530,671	99.98
TR187	2011.10.08	Hospital A	Outbreak	PT1-1	ST11	5114610	5,535,384	99.98
TRqt-44	2011.10.14	Hospital A	Outbreak	PT1-1	ST11	1929825	5,550,893	99.99
TR191	2011.10.10	Hospital A	Outbreak	PT1-1	ST11	4776142	5,547,137	99.99
TRqt-38	2011.10.11	Hospital A	Outbreak	PT1-1	ST11	2313187	5,552,446	99.99
TR215	2011.11.01	Hospital A	Outbreak	PT1-1	ST11	5113987	5,529,661	99.99
TR249	2011.11.28	Hospital A	Outbreak	PT1-1	ST11	5070157	5,530,509	99.98
TR237	2011.11.22	Hospital A	Outbreak	PT1-1	ST11	4923904	5,551,691	99.99
TR213	2011.11.02	Hospital A	Outbreak	PT1-1	ST11	4889624	5,535,549	99.98
TR214	2011.10.31	Hospital A	Outbreak	PT1-1	ST11	4433786	5,546,545	99.99
TR210	2011.10.24	Hospital A	Outbreak	PT1-1	ST11	5506132	5,539,211	99.99
TR197	2011.10.11	Hospital A	Outbreak	PT1-1	ST11	5208391	5,551,279	99.99
TRqt-37	2011.10.11	Hospital A	Outbreak	PT1-1	ST11	5368038	5,541,521	99.99
TRqt-47	2011.10.18	Hospital A	Outbreak	PT1-5	ST11	1210367	5,476,896	99.99
TRqt-48	2011.10.18	Hospital A	Outbreak	PT1-1	ST11	1986985	5,502,861	99.98
TR198	2011.10.16	Hospital A	Outbreak	PT1-4	ST11	1498670	5,544,398	99.99
TR231-M	2011.11.19	Hospital A	Outbreak	PT1-6	ST11	2002247	5,714,983	99.99
TR221	2011.11.16	Hospital A	Outbreak	PT1-6	ST11	1981562	5,722,498	99.99
TRqt-43	2011.10.18	Hospital A	Outbreak	PT1-1	ST11	2222462	5,553,936	99.99
TR258	2011.12.12	Hospital A	Outbreak	PT1-2	ST11	4825797	5,788,988	99.99
TRqt-41	2011.10.18	Hospital A	Outbreak	PT1-1	ST11	5272726	5,550,545	99.99
TR200	2011.10.21	Hospital A	Outbreak	PT1-1	ST11	3560444	5,546,753	99.99
TR209	2011.10.24	Hospital A	Outbreak	PT1-1	ST11	4717301	5,549,132	99.99
TRqt-49	2011.10.27	Hospital A	Outbreak	PT1-1	ST11	1997590	5,545,007	99.98
TR207	2011.10.29	Hospital A	Outbreak	PT1-1	ST11	5510939	5,550,137	99.99
TR262	2011.12.23	Hospital A	Outbreak	PT1-3	ST11	2551943	5,574,152	99.98
A1502	2012.08.17	Hospital B	Sporadic	PT3	ST11	4735099	5,352,016	99.98
A1732	2011.12.13	Hospital C	Sporadic	PT6	ST11	3006404	5,361,926	99.99
A1760	2011.12.19	Hospital D	Sporadic	PT2	ST11	6285811	5,558,188	99.99
A1763	2011.12.2	Hospital E	Sporadic	PT4	ST11	3486502	5,473,965	99.99
A1771	2011.12.22	Hospital F	Sporadic	PT5	ST11	3094363	5,712,663	99.99

The newly sequenced strains were stored at −70°C in brain heart broth with 20% sterile glycerol in our lab. All strains were routinely cultured on Mueller-Hinton (MH) agar plates, and typical colonies were picked up, identified by biochemical tests using the API®-20E test kits (bioMérieux, Lyon, France). The bacteria were grown at 37°C for 18–24 h for preparation of DNA extraction using the QIAampDNA Mini kit (Qiagen, Hilden, Germany) according to the manufacturer's instructions.

### Whole-genome sequencing and assembly

Bacterial strains were sequenced using an Illumina GA IIx (Illumina Inc., San Diego, CA, USA) by constructing two paired-end (PE) libraries with average insertion lengths of 500 and 2,000 bp. Raw data was processed in four steps, including removing reads with 5 bp of ambiguous bases, removing reads with 20 bp of low quality (≤Q20) bases, removing adapter contamination, and removing duplicated reads. Finally, 100× libraries were obtained with clean paired-end read data. Assembly was performed using SOAPdenovo v2.04 (Li et al., 2010).

### cgMLST target gene definition

To screen the candidate genomes used to identify cgMLST target gene set, a total of 907 whole genomes of *K. pneumoniae* obtaining from the NCBI database were used. The genomes were filtered if they met the following criteria: (i) genomes that with contig number ≥200, (ii) genomes that don't contain all seven MLST genes or with multiple copies (identity ≥90%, overlap = 100%), and (iii) genomes that having <3,000 single copy homologous genes of candidate target genes.

To screen the candidate cgMLST target genes, the complete genome of *K. pneumoniae* subsp. pneumoniae strain HKUOPLC (GenBank assembly accession number GCA_001280925.1) was used and these parameters comprise the following filters to exclude certain genes of reference genome from the cgMLST scheme: (i) a minimum length filter that discards all genes shorter than 50 bp; (ii) a start codon filter that discards all genes that contain no start codon at the beginning of the gene; (iii) a stop codon filter that discards all genes that contain no stop codon or more than one stop codon or that do not have the stop codon at the end of the gene; (iv) a homologous gene filter that discards all genes with fragments that occur in multiple copies within a genome (with identity of 90% and >100 bp overlap); and (v) a gene overlap filter that discards the shorter gene from the cgMLST scheme if the two genes affected overlap >4 bp. Furthermore, the plasmid and transposon gene filter were performed as following: (i) filter genes that is highly homologous with 126 *Klebsiella* plasmid genomes (with identity >90%, overlap >95%); (ii) filter genes that is homologous with transposon_db TransposonPSI database (with identity >50%, coverage >70%).

The remaining genes were then used in a pairwise comparison with BLAST version 2.2.12 (parameters used were word size 11, mismatch penalty −1, match reward 1, gap open costs 5, and gap extension costs 2) with the query *K. pneumoniae* chromosomes. All genes of the reference genome that were common in all query genomes with a sequence identity of ≥90% and 100% overlap and, with the default parameter stop codon percentage filter turned on, formed the final cgMLST scheme; this discards all genes that have internal stop codons in > 20% of the query genomes.

### Nucleotide sequence accession number

This Whole Genome Shotgun project has been deposited at GenBank under the Bioproject ID PRJNA313004, accession LUVP00000000-LUGR00000000, MAYI00000000-MAYL00000000, MBNA00000000-MBNO00000000, MBNQ00000000-MBMZ00000000.

## Results

### Development of a cgMLST scheme

**Figure 2:**
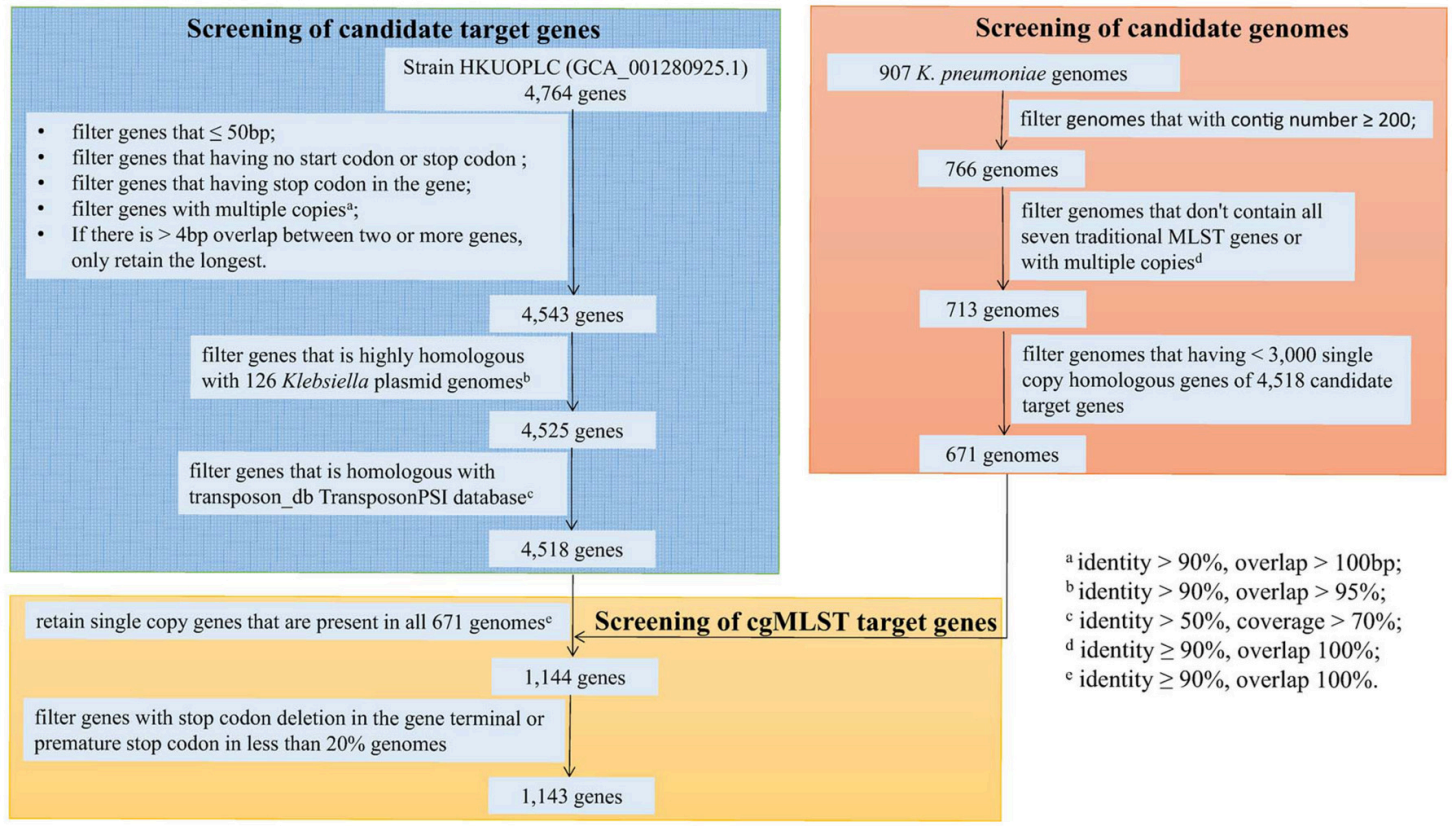
**Flowchart to determine cgMLST target genes**.

Using *K. pneumoniae* strain HKUOPLC (GenBank assembly accession number GCA_001280925.1) as the reference genome (4,764 genes), after basic filter based on minimum length, start codon, stop codon, homologous gene and gene overlap, 4,543 genes were retained. Further filtering of plasmid and transposon gene had removed 18 and 7 genes respectively. So there were 4,518 genes being retained as cgMLST candidate target gene set (Figure 2).

A total of 907 genomes of *K. pneumoniae* were download from NCBI database and used as original genome dataset for candidate genomes screening. Firstly, 141 genomes that with contig number more than 200 were filtered; secondly, 53 genomes that don't contain all the seven traditional MLST gene fragments or contain multiple copies of traditional MLST genes were filtered; finally, 42 genomes that having < 3,000 single copy homologous genes of 4,518 candidate genes were filtered. So 671 genomes were selected as candidate genomes set for screening of cgMLST target genes (Figure 2).

**Figure 3:**
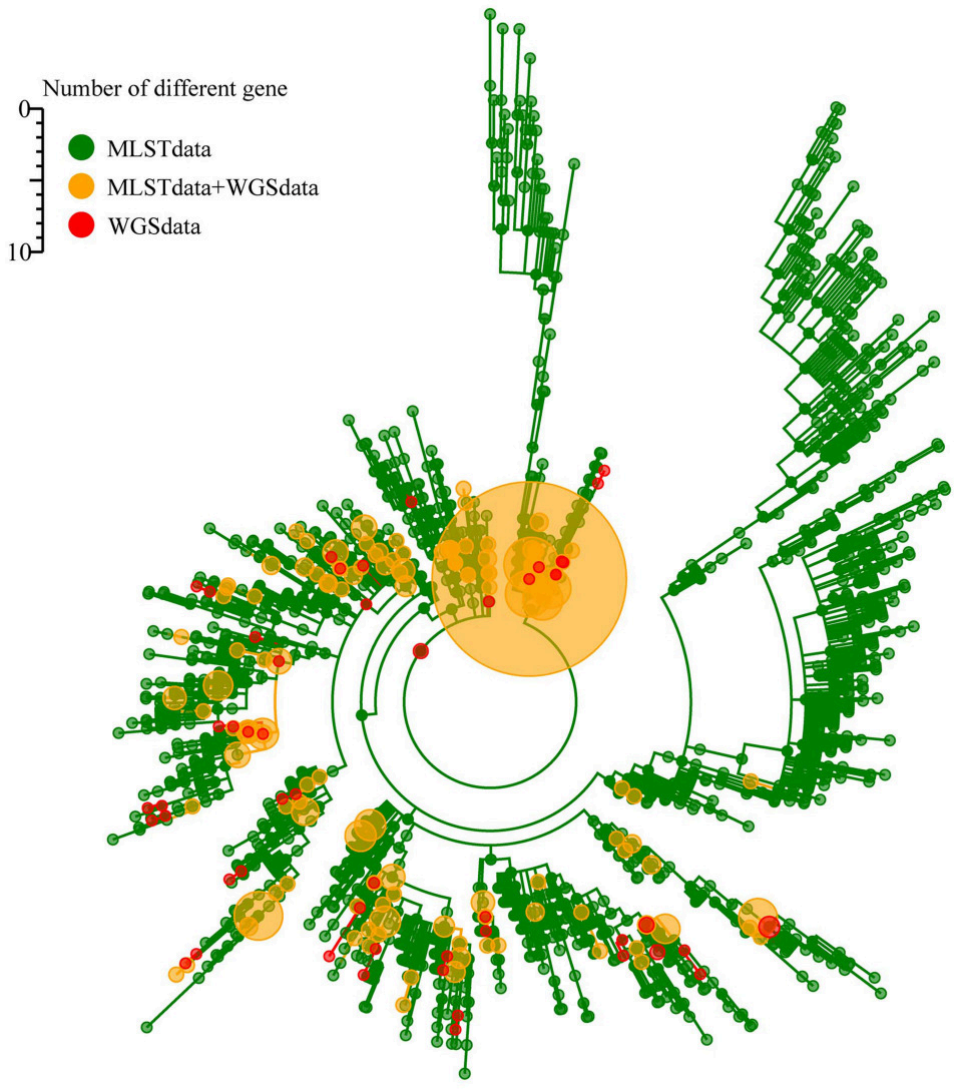
**The representation of 671 genomes of ***K. pneumoniae*** population based on the K. pneumoniae MLST database**.

The sequences of the seven traditional MLST genes comprising the allelic profile of the traditional MLST scheme were extracted separately from the genome sequences and queried against the *K. pneumoniae* MLST database. A minimum spanning tree was structured based on the allelic profile of 671 genomes and 1989 STs in the *K. pneumoniae* MLST database (1st March, 2016). In the minimum spanning tree, the 671 genomes distributed in the whole tree, suggest the dataset of 671 genomes having a good representation of *K. pneumoniae* population (Figure 3).

**Figure 4:**
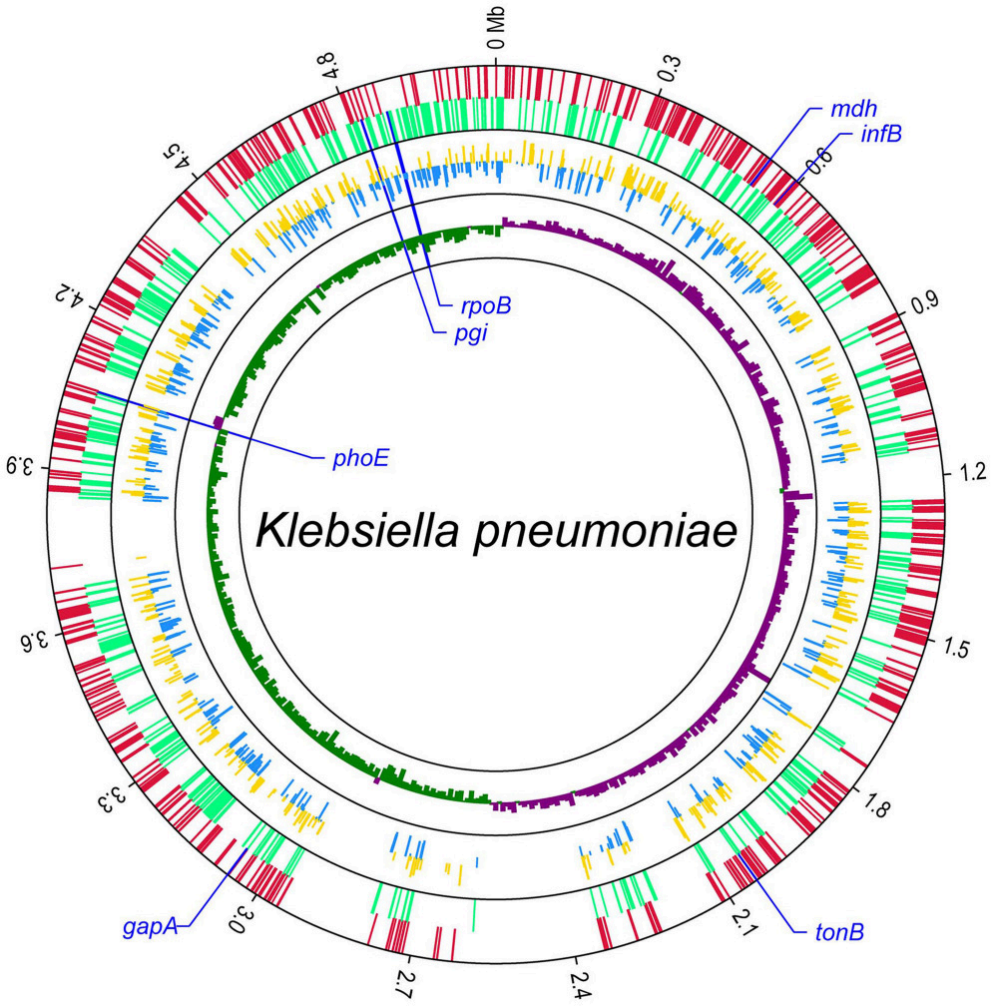
**Distribution of 1,143 cgMLST target genes within the genome of ***K. pneumoniae*** subsp. pneumoniae strain HKUOPLC**. The GenBank assembly accession number of HKUOPLC is GCA_001280925.1. Outer circle, cgMLST target genes (red: the genes in positive strand; light green: the genes in negative strand.); middle circle, allele number of cgMLST target genes (yellow: allele number of genes in positive strand; blue: allele number of genes in negative strand.); GCskew value (ratio of G-C/G+C) (violet: GCskew value of the genes in positive strand; bottle green: GCskew value of the genes in negative strand.) The locations of seven traditional MLST genes were special marked.

Then the 671 genomes were used to screen out cgMLST target gene set from the 4,518 candidate target genes. There were 1,144 genes of the 4,518 candidate target genes were common in all 671 genomes with a sequence identity of ≥90% and 100% overlap. Furthermore, one gene had internal stop codons in >20% of the 671 genomes and was discarded. So a core genome was defined as a standard set of 1,143 genes (24.0% of the whole reference genome) for the cgMLST scheme. These 1,143 cgMLST target genes were randomly distributed across the genome (Figure 4).

### Retrospective analysis of nosocomial infection outbreak strains

**Figure 5:**
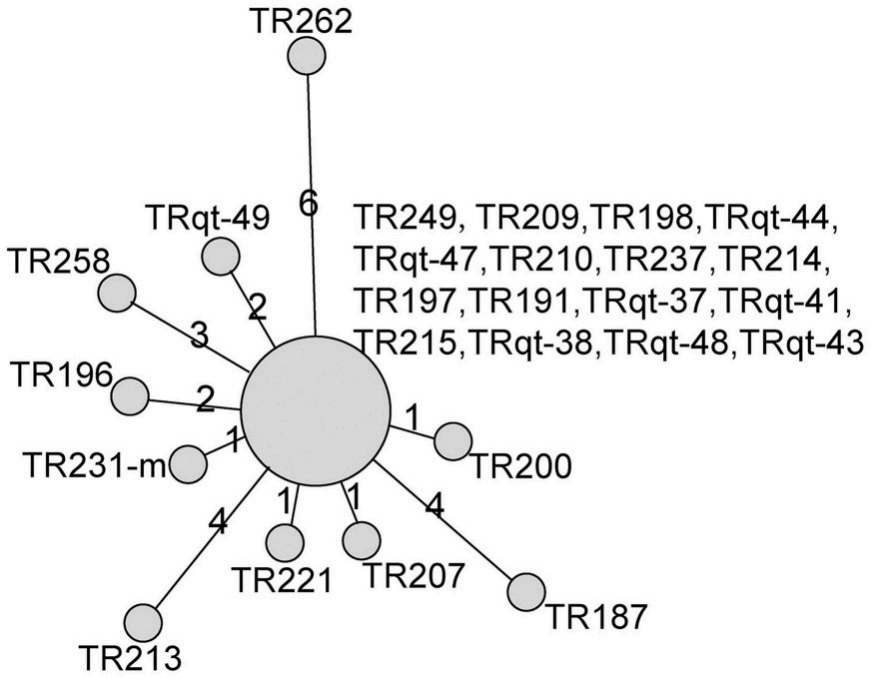
**Minimum-spanning tree based on cgMLST allelic profiles of 26 ***K. pneumoniae*** isolates from SICU outbreak and five outgroup strains**. The five outgroup strains were A1502, A1732, A1760, A1763, and A1771. Each circle represents an allelic profile based on sequence analysis of 1,143 cgMLST target genes. The numbers on the connecting lines illustrate the numbers of target genes with differing alleles. The different groups of strains are distinguished by the colors of the circles.

The cgMLST scheme was then used to analyze isolates from a nosocomial *K. pneumoniae* infection outbreak. The outbreak occurred in a surgical intensive care unit (SICU) of a general hospital in Beijing between October and December, 2011. Epidemiological investigation and molecular subtyping (PFGE and MLST) results confirmed that this was a nosocomial infection outbreak caused by epidemic KPC-2-producing ST11 *K. pneumoniae* clone (Table 1, Figure 5).

For the retrospective analysis, the 26 SICU outbreak strains were divided into 11 cgMLST types. There was a dominant cgMLST type containing 16 strains and other SICU strains showed one to six allelic differences to the dominant cgMLST type. The maximum allelic distance within the outbreak was 10 alleles. Extraction of classical MLST targets resulted in STs of all isolates that were identical to those of ST11 and confirmed the Sanger sequencing (Table 1). In comparison, the other ST11 strains showed 23 or more allelic differences to the SICU strains.

### cgMLST typing of ST11 strains

ST1 is the most prevalent ST of the carbapenem-resistant *K. pneumoniae* isolates in China and also one of the predominant clones worldwide. Twenty-two epidemiological unrelated ST11 strains were put together with SICU outbreak strains to evaluate for the discriminatory power of cgMLST typing. Among the epidemiological unrelated strains, eight were isolated from China, eight from the United States of America (USA), six from European countries and one from Israel.

**Figure 6:**
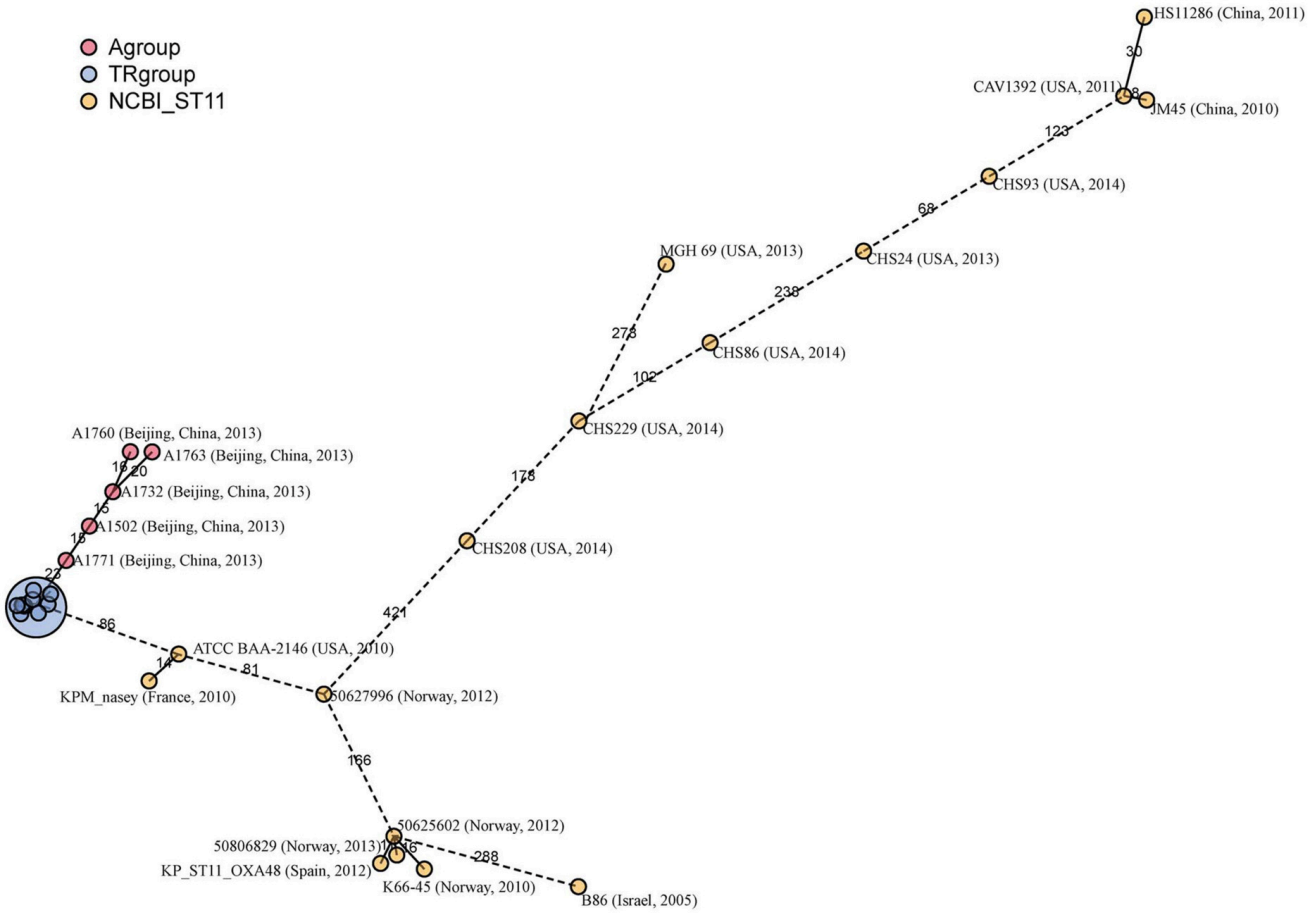
**Minimum-spanning tree based on cgMLST allelic profiles of 23 ST11 strains**. The 23 strains contained SICU outbreak strains (as one strain in this tree), five ST11 isolates (A1502, A1732, A1760, A1763 and A1771) newly sequenced in this study and 17 isolates whose whole genome sequences were obtained from the NCBI database. Each circle represents an allelic profile based on sequence analysis of 1,143 cgMLST target genes. The numbers on the connecting lines illustrate the numbers of target genes with differing alleles. The different groups of strains are distinguished by the colors of the circles.

In the minimum-spanning tree, cgMLST correctly grouped all SICU strains together with a maximum of 10 allelic differences (Figure 6). However, there were two clusters of non-outbreak strains with allelic differences less than 10. The first cluster contained two strains from China and USA respectively and the second cluster contained two strains from Norway. By used 30 allelic differences to be as the criteria of a cluster, there were four clusters and each cluster contained strains from different countries. The strains outside of the four clusters showed at least 68 allelic differences.

## Discussion

An accurate, rapid, and standardized subtyping of bacterial isolates is required in outbreak investigation and population structure analysis. MLST has been one of the most widely used bacterial pathogen subtyping techniques since it was developed in the late 1990s (Maiden et al., 1998). A major advantage of MLST is the easily storage and management allelic data in a central database and comparison data from different laboratories using universal nomenclatures. In this study, we developed a WGS-based MLST typing method, named cgMLST, for *K. pneumoniae* subtyping. cgMLST analyzes hundreds of genes using next-generation sequencing, which dramatically increases discriminatory power. Furthermore, we applied this approach to one *K. pneumoniae* outbreak and population structure analysis of epidemic ST11 clone. The cgMLST typing scheme developed in this study is able not only to differentiate outbreak from non-outbreak *K. pneumoniae* isolates clearly but also to reveal that there may be several sub-clones of epidemic ST11 clone.

In a previous study, a set of 634 genes were defined as the cgMLST set by comparison of 167 *K. pneumoniae* genome sequences by the researchers of Institut Pasteur, Paris, France and other institutes (Bialek-Davenet et al., 2014). Cluster analysis based on these 634 genes enabled precise definition of globally distributed hypervirulent and multidrug-resistant clonal groups. Furthermore, the Pasteur-cgMLST scheme showed good epidemiologic relevance in outbreak investigation of clonal group 258 strains (Bialek-Davenet et al., 2014; Onori et al., 2015). There were several other clusters of *K. pneumoniae* infection have been investigated to date using a WGS-based approach (Snitkin et al., 2012; Lee et al., 2014; Jiang et al., 2015; Marsh et al., 2015; Mathers et al., 2015; Onori et al., 2015; Zhou et al., 2016). Among these studies, two used cgMLST methods and yielded comparable results to that of WGS-based SNP (Snitkin et al., 2012; Onori et al., 2015). One study provided a clonality analysis of a KPC-producing *K. pneumoniae* strain isolated in Korea by both WGS-based SNP and cgMLST, and both methods supported the closer relatedness of different strains carrying *bla*_*KPC*_ genes (Snitkin et al., 2012). Another study provided an investigation of nosocomial *K. pneumoniae* infections and outbreaks, and suggested that the cgMLST tree is largely consistent with the tree resulting from the SNP-based phylogenetic analysis (Onori et al., 2015). In this study, we used 907 whole genomes as original genome dataset for cgMLST scheme developing and 671 whole genomes were screened out to screen cgMLST target genes. These strains were isolated from all parts of the world and represented the population of *K. pneumoniae* as showed by traditional MLST. However, all these studies were difficult to reproduce and to compare, especially given the differences in reference genomes and bioinformatics pipelines used, as well as software parameter selections. So it is required that establishing an web-based nomenclature server that can be used to query, compare and analyze data like the current MLST servers for any user worldwide.

Several criteria have been proposed for evaluating the performance of typing systems in outbreak investigation, including typeability, reproducibility, discriminatory power, and epidemiologic concordance (Struelens, 1998). For cgMLST, the typeability and reproducibility must be a really good as it is based on sequencing. So the discriminatory power and epidemiologic concordance are the two most important criteria for cgMLST evaluation. In future studies, more strains with clear epidemiological and genetic background showed be analyzed to establish the criteria for outbreak investigations and to further evaluate the discriminatory power and epidemiologic concordance of cgMLST.

PFGE is considered the gold standard of molecular typing methods for outbreak investigation because of its high degree of discriminatory power (Struelens, 1998). In this study, both PFGE and cgMLST produced dominant types of the outbreak strains. However, the strains of the PFGE and cgMLST dominant types were not exactly the same. This may be explained that these two subtyping methods examine and distinguish different types of genetic mutation events. For PFGE, there is a rule established by Tenover and colleagues to interpret the differences of PFGE patternsin outbreak investigations (Tenover et al., 1995). By Tenover et al.'s criterion, the strains with 0, 1–3, 4–6, and ≥7 fragment differences to outbreak pattern suggested indistinguishable, closely related, possibly related and different to outbreak strain, respectively. This criterion is useful in short-term outbreak investigations, especially for nosocomial outbreaks and foodborne outbreaks. So a general or a species specific criterion for interpretation of differences in cgMLST profiles is needed for outbreak investigations.

Within the outbreak investigated here, very few allelic changes of cgMLST were detected and the maximum allelic distance within the outbreak was only 10 alleles. This high similarity reflects the outbreak situation without much time for intraoutbreak microevolution, because all patients were hospitalized in the same hospital during period less than 2 months. In previous studies, a maximum of allelic differences ≤10 were observed within an outbreak caused by *L. monocytogenes* (Ruppitsch et al., 2015), *M. tuberculosis* (Kohl et al., 2014), and *L. pneumophila* (Moran-Gilad et al., 2015). So in the using of cgMLST for outbreak investigation, the cluster type threshold of ≤10 differences warrants further comments. However, more studies are needed to define parameters for cgMLST in outbreak investigations, molecular epidemiological and population structure studies.

In conclusion, we devised a cgMLST scheme for WGS-based typing of *K. pneumoniae*, which showed satisfactory discriminatory power for analysis of outbreak *K. pneumoniae* strains and revealing the in-depth population structure of clone groups defined by traditional 7-loci MLST. cgMLST thus has the potential for becoming a gold standard tool for *K. pneumoniae* subtyping. The remaining challenge is to establish an Internet-based nomenclature server, like EnteroBase (http://enterobase.warwick.ac.uk/), which can be used to facilitate universal global nomenclature for any user.

## Author contributions

HZ designed the study, carried out the experiments and the data analysis and wrote the manuscript. WL and CL performed the bioinformatic analysis. TQ and HR participate in the experiments and the data analysis.

### Conflict of interest statement

The authors declare that the research was conducted in the absence of any commercial or financial relationships that could be construed as a potential conflict of interest.
